# Practical Digital Competence and Psychosocial Well-Being Among Adults with Severe Disabilities: Cross-Sectional Indirect Associations Through Perceived Self-Determination in Daily Life

**DOI:** 10.3390/healthcare14182897

**Published:** 2026-09-08

**Authors:** Hyun Namgung, Moon-June Oh

**Affiliations:** 1 Dharma College, Dongguk University, Seoul 04620, Republic of Korea; 2 Department of Policy Research, Seoul Welfare Foundation, Seoul 04147, Republic of Korea; miccross@welfare.seoul.kr

**Keywords:** self-reported practical digital competence, psychosocial well-being, adults with severe disabilities, perceived self-determination, subjective social isolation, cross-sectional indirect association, digital health equity, digital inclusion, digital divide

## Abstract

**Highlights:**

**What are the main findings?**

- Among adults with severe disabilities in Seoul (*N* = 1519), higher self-reported practical digital competence was associated with greater psychosocial well-being in cross-sectional analyses.
- The indirect association through perceived self-determination in daily life was statistically significant across the main and sensitivity analyses, whereas evidence for an indirect association through subjective social isolation was limited and specification-sensitive.

**What are the implications of the main findings?**

- Digital inclusion programs for adults with severe disabilities may benefit from supporting practical digital skills relevant to everyday activities rather than focusing only on device access or connectivity.
- The findings suggest the potential relevance of practical digital skills and perceived autonomy in daily life as complementary components of digital inclusion efforts. Subjective social disconnection may require broader relational and environmental supports; these findings are cross-sectional and do not establish temporal or causal relationships.

**Abstract:**

**Background/Objectives**: Digital inequalities are increasingly recognized as a health-equity issue, yet self-reported practical digital competence and psychosocial well-being among adults with severe disabilities remain understudied. This study examined their cross-sectional association and indirect associations through perceived self-determination in daily life and subjective social isolation in a parallel model. **Methods**: Cross-sectional data from 1519 adults in a 2023 Seoul survey were analyzed. Self-reported practical digital competence was assessed with six task-ability items, perceived self-determination in daily life with one item, and subjective social isolation with two items. Adjusted product-of-coefficients models used 2000 bootstrap replications to calculate bias-corrected confidence intervals. **Results**: Self-reported practical digital competence was positively associated with psychosocial well-being (b = 0.085, *p* < 0.001). The indirect association through perceived self-determination in daily life was significant in the main analysis (estimate = 0.076, BC 95% CI [0.050, 0.106]) and remained significant across all sensitivity analyses. Self-reported practical digital competence was not significantly associated with subjective social isolation (b = −0.026, *p* = 0.203), and the corresponding indirect association was nonsignificant (estimate = 0.006, BC 95% CI [−0.003, 0.016]) and inconsistent across specifications. The direct association was near zero and nonsignificant after both psychosocial variables were included. **Conclusions**: The indirect association through perceived self-determination in daily life was consistent across analyses, but this single-item measure should be interpreted as a narrow indicator of perceived autonomy or control rather than the broader multidimensional construct of self-determination. Evidence for subjective social isolation was limited and specification-sensitive, and simultaneous measurement precludes temporal or causal interpretation. Digital inclusion efforts may benefit from combining practical digital skills with opportunities to support meaningful goals and everyday choices. Broader relational and environmental supports may also be needed for subjective social disconnection, and longitudinal and intervention research is warranted.

## 1. Introduction

Health information, welfare services, and public administration are increasingly digitalized, transforming the environments through which individuals access healthcare, manage daily needs, and participate in social and institutional life [1,2]. Digital inequalities—encompassing disparities in access to devices, connectivity, digital skills, and usable digital environments—are therefore increasingly recognized as a meaningful dimension of health equity [1,3]. Frameworks attentive to the digital determinants of health emphasize that meaningful digital inclusion requires access to digital infrastructure, competence in navigating digital environments effectively, and accessible and usable digital service design [2,3,4]. As health and welfare delivery shifts toward digital platforms, the ability to engage with these environments may have implications for access to care, use of social supports, and participation in daily life [1,2,3,4].

Persons with disabilities commonly experience compounded barriers to healthcare access, community participation, and independent living, including functional limitations, mobility constraints, support needs, and accessibility barriers in physical and social environments [5,6,7,8]. Digitalization may both open new avenues for participation—by enabling remote access to services, health information, and social communication—and introduce new forms of exclusion for those who face barriers to effective digital engagement [9]. Adults with severe disabilities warrant specific attention because greater functional and support needs may increase both the potential value of digital tools and the consequences of inaccessible or unusable digital environments; limited digital competence may further constrain their access to increasingly digitalized health and welfare systems [5,6,7,8,9]. Psychosocial well-being—encompassing subjective dimensions of happiness, life satisfaction, and perceived meaning—is an important subjective dimension of quality of life in this population [10]. In South Korea, where public health and welfare services are increasingly mediated through digital platforms, these digital conditions may be especially salient for adults with severe disabilities [11].

Despite the importance of distinguishing digital access from the practical ability to use digital technologies in this population, prior research has often relied on indicators such as device ownership, internet access, and frequency of internet use [12,13,14,15]. These indicators provide useful information about access and engagement but do not directly indicate whether individuals can perform practical digital tasks relevant to daily life management and service navigation. Self-reported practical digital competence—understood here as respondents’ perceived ability to perform specific tasks on personal computers and mobile devices—represents a task-specific indicator of digital capability that may be more directly relevant to daily life engagement than access-based measures alone [16,17]. As operationalized here, however, it reflects perceived task ability rather than objectively demonstrated performance. Prior studies among persons with disabilities have linked digital access or internet use to well-being and mental health [9,15,18], but evidence specifically examining task-specific digital competence—particularly among adults with severe disabilities—remains limited. The present operationalization captures a practical, task-specific dimension of perceived digital ability but does not encompass all aspects of digital engagement, including health-specific digital tasks or assistive technology use.

The association between self-reported practical digital competence and psychosocial well-being may be examined in relation to psychosocial factors that are theoretically associated with both digital engagement and well-being. Research grounded in disability-specific self-determination and quality-of-life frameworks has emphasized the centrality of autonomy, choice, and self-directed participation in shaping quality of life and well-being among people with disabilities [19,20,21,22,23]. For adults with severe disabilities, whose opportunities for independent participation may be constrained by functional limitations and structural barriers, the ability to use digital tools to manage everyday tasks may be associated with a greater sense of perceived control and self-direction in daily life [19,20,23]. These theoretical considerations motivate examination of a cross-sectional indirect-association pattern involving perceived self-determination in daily life. In the present study, perceived self-determination in daily life is operationalized using a single survey item and is therefore treated as a narrow subjective indicator of perceived freedom and control in everyday life rather than as a measure of the full multidimensional construct of self-determination. Accordingly, it is hypothesized that self-reported practical digital competence would be positively associated with perceived self-determination in daily life (H1), and that perceived self-determination in daily life would in turn be positively associated with psychosocial well-being (H2).

Subjective experiences of social disconnection, including loneliness, are significant concerns for adults with severe disabilities, who may face reduced opportunities for social interaction due to mobility limitations, accessibility barriers, and constraints on community participation [24]. Digital communication tools may support social connectedness among people with disabilities [9], but evidence linking digital technology use to loneliness or subjective social disconnection remains mixed, and any social benefits may depend on interaction quality, pre-existing social networks, and broader contextual factors [25]. Despite the mixed evidence regarding digital engagement and subjective social disconnection, subjective social isolation remains an important psychosocial factor because it has been consistently associated with poorer mental health and psychosocial well-being across populations [24,26]. The construct is termed subjective social isolation in this study because the available items reflect perceived loneliness and social disconnection rather than objective network size, contact frequency, or community participation. It is therefore hypothesized that self-reported practical digital competence would be negatively associated with subjective social isolation (H3), and that subjective social isolation would in turn be negatively associated with psychosocial well-being (H4). Given the mixed evidence regarding digital engagement and subjective social isolation, however, this indirect-association pattern was specified as theoretically plausible but less certain a priori.

Perceived self-determination in daily life and subjective social isolation were selected as two complementary, rather than exhaustive, psychosocial dimensions because they capture theoretically distinct aspects of psychosocial experience—one reflecting perceived personal autonomy and control in daily life, and the other reflecting subjective social disconnection. They were specified as parallel psychosocial variables because existing theory and evidence did not establish a clear temporal ordering between them, and the cross-sectional data could not establish such an ordering. The model was therefore intended to compare their simultaneous indirect-association patterns rather than to test a serial process.

Relatively few studies have examined the association between self-reported practical digital competence and psychosocial well-being among adults with severe disabilities, and fewer still have considered perceived self-determination in daily life and subjective social isolation as parallel psychosocial variables within the same analytical framework. The present study extends prior research in three respects: it focuses on self-reported ability to perform specific PC and mobile tasks rather than indicators of access or frequency of use alone; examines registered adults with severe disabilities, for whom the opportunities and barriers associated with digitalization may be especially consequential; and compares two theoretically distinct dimensions of psychosocial experience within the same cross-sectional model.

**Figure 1:**
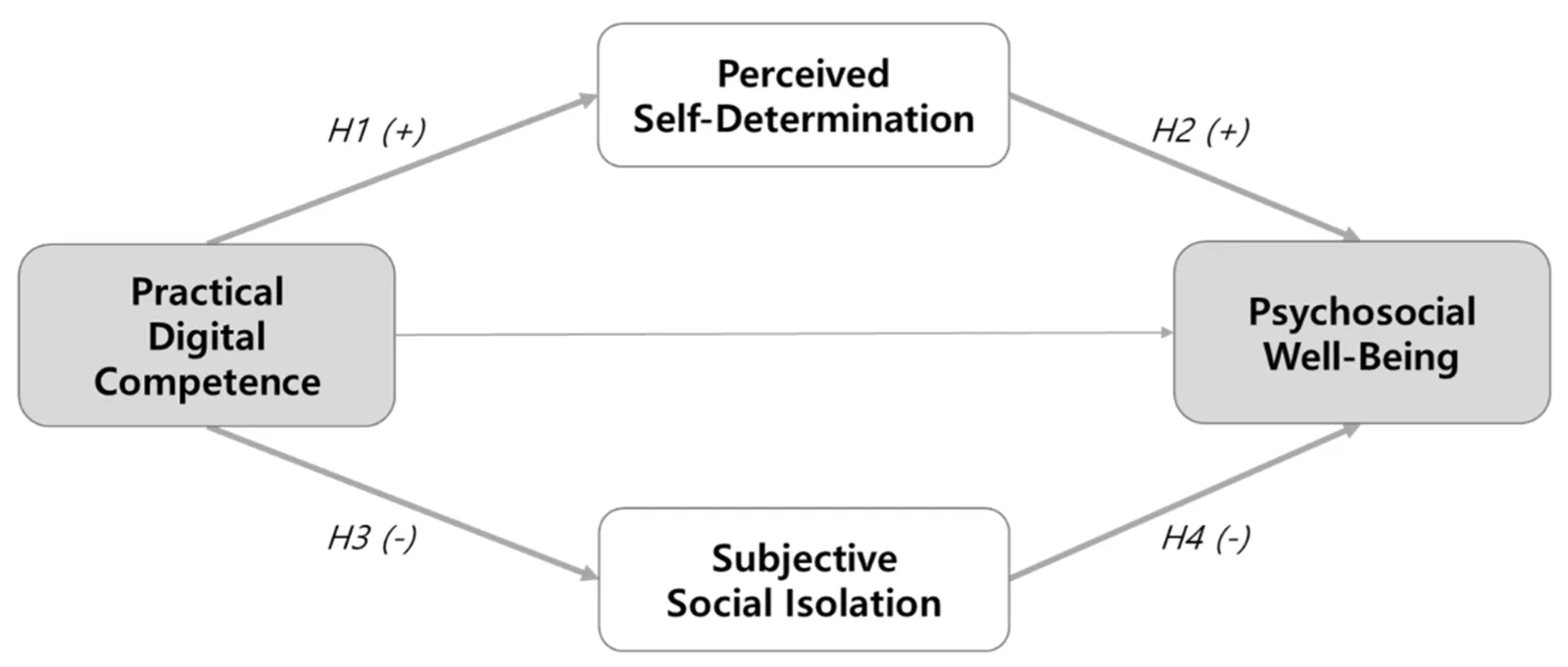
Conceptual model of cross-sectional associations. Note: H1–H4 represent the hypothesized associations. The direct association between self-reported practical digital competence and psychosocial well-being was estimated without a directional hypothesis. The ordering shown in the model is theoretically specified and does not imply temporal or causal relationships.

To address these gaps, the present study draws on data from the 2023 Survey on the Actual Conditions of Independent Living of People with Disabilities in Seoul, South Korea (*N* = 1519), to examine the cross-sectional association between self-reported practical digital competence and psychosocial well-being and to estimate the indirect associations through perceived self-determination in daily life and subjective social isolation. Because all variables were measured at the same time, the ordering represented in the model is theoretically specified rather than temporally established, and reverse or reciprocal associations remain plausible. The direct association between self-reported practical digital competence and psychosocial well-being is also estimated within this framework, although no directional hypothesis is advanced for this path. Figure 1 depicts the hypothesized associations from self-reported practical digital competence to each psychosocial variable, the associations from each psychosocial variable to psychosocial well-being, and the direct association between self-reported practical digital competence and psychosocial well-being estimated after accounting for both psychosocial variables.

## 2. Materials and Methods

### 2.1. Data and Study Population

This study analyzed data from the 2023 Survey on the Actual Conditions of Independent Living of People with Disabilities in Seoul, conducted jointly by the Seoul Metropolitan Government and the Seoul Welfare Foundation [27]. The survey was designed to assess the living conditions, independent living experiences, welfare needs, health-related circumstances, social relationships, digital access, and subjective well-being of persons with severe disabilities residing in Seoul, South Korea.

The target population comprised community-dwelling adults aged 18 years or older with registered severe disabilities. In the Korean disability registration system, severe disability corresponds to what was classified as disability grades 1–3 under the prior six-grade severity scale, which has since been restructured into severe and mild disability categories. Because disability severity is determined separately within each disability type, severity classifications are not directly comparable across disability categories. The sampling frame was constructed from the national disability registry maintained by the Ministry of Health and Welfare as of 31 December 2022. Thus, the survey population represents registered, community-dwelling adults with severe disabilities residing in Seoul and excludes unregistered individuals, institutional residents, and people residing outside Seoul.

A stratified systematic sampling design was used, with stratification by disability type, sex, and age. To improve representation of smaller disability-type and age strata, disproportionate allocation procedures were used, including minimum allocation within strata and square-root proportional allocation for selected strata. Post-stratification weights were included in the original dataset and were constructed from inverse selection probabilities with nonresponse adjustments and post-stratification by disability type, sex, and age group [27].

Data were collected between August and September 2023 through face-to-face interviews conducted by trained interviewers using Tablet-Assisted Personal Interviewing (TAPI). The TAPI system incorporated real-time validation checks to minimize data entry errors. Response assistance was recorded in the survey. A total of 952 respondents (62.7%) completed the interview without assistance, while 175 (11.5%) received partial assistance and 392 (25.8%) received full assistance from another person identified in the survey as a guardian or other support person. Because the dataset does not indicate whether the assisting person independently provided responses or supported the respondent in communicating their own responses, these cases are described as assisted interviews rather than proxy interviews. The initial target sample size was 1500 respondents; after fieldwork, field verification, and data quality control, 1519 valid completed interviews were retained in the finalized dataset, slightly exceeding the survey target [27]. Of these 1519 valid completed interviews, no respondents were excluded because of missing data on the outcome, psychosocial variables, or covariates; therefore, all 1519 respondents were retained in the analytic sample.

All 1109 ICT device owners had complete responses to the six digital-competence items. Among the 410 respondents who reported not owning the relevant ICT devices, the six digital-competence items were skipped by survey design. These unadministered items therefore represented structural skips rather than item nonresponse and were not statistically imputed. They were handled according to the coding rule described in Section 2.2, with alternative coding and device-owner-only specifications examined in sensitivity analyses. No listwise deletion was applied.

Institutional review board review was not required for the present study under the relevant Korean regulations. The data were collected by a public authority for the purpose of evaluating public welfare programs. Under Article 2, Paragraph 2 of the Enforcement Rule of the Bioethics and Safety Act of the Republic of Korea, studies conducted or commissioned by national or local governments to review or evaluate public welfare or service programs are not classified as human subjects research requiring such review [28]. The present study involved the secondary analysis of anonymized data, and the authors did not recruit participants or conduct the original data collection. The original survey implemented standardized participant-information and consent procedures before the interviews.

### 2.2. Measures

All study variables were constructed from items included in the survey [27]. All focal measures were derived from survey responses collected during the same interview; no performance-based or externally assessed measures were available. The analytic framework included one outcome variable, one key independent variable, two psychosocial variables, and a set of covariates. Unless otherwise noted, higher scores indicate higher levels of the construct being measured.

#### 2.2.1. Outcome Variable: Psychosocial Well-Being

Psychosocial well-being was measured using three items that captured positive subjective evaluations of daily life: happiness experienced on the previous day, overall life satisfaction, and the perceived value of the things one does. Respondents rated each item on a five-point scale (1–5). The original responses were reverse-coded so that higher values indicated greater psychosocial well-being. A composite score was calculated as the mean of the three items. The scale demonstrated high internal consistency (Cronbach’s α = 0.90).

#### 2.2.2. Key Independent Variable: Self-Reported Practical Digital Competence

Self-reported practical digital competence was operationalized using six items assessing respondents’ perceived ability to perform everyday digital tasks on personal computers (PCs) and mobile devices. Three items assessed PC-related competence: connecting and using various external devices, such as digital cameras, printers, scanners, and USB storage devices; transmitting files from a PC to others via the internet; and creating documents on a PC. The other three items assessed mobile-device competence: changing device settings, transferring files or data, and creating documents. Each item was rated on a four-point scale (1–4). The original responses were reverse-coded so that higher values indicated greater self-reported competence. The items assessed respondents’ perceived task ability rather than performance demonstrated under standardized conditions.

Among the 1109 respondents who reported owning ICT devices, self-reported practical digital competence was calculated as the mean of the six items. Among device owners, the six-item scale demonstrated very high internal consistency (Cronbach’s α = 0.97). The three-item PC and mobile-device subsets also demonstrated high internal consistency (Cronbach’s α = 0.98 and 0.96, respectively). In the main specification, the 410 respondents who did not own ICT devices and for whom the six competence items were structurally skipped were assigned the minimum score of 1 as an analytic coding rule to retain non-owners in the full-sample analysis. This assignment was not statistical imputation and should not be interpreted as evidence that non-owners necessarily had the lowest underlying digital competence. Because task-specific competence on the relevant devices could not be directly assessed without device access, the primary specification may partly capture both device access and self-reported task ability. Because this coding decision could affect the estimated association between self-reported practical digital competence and psychosocial well-being, sensitivity analyses using alternative specifications were conducted, as described in Section 2.3 and reported in Section 3.3.

#### 2.2.3. Psychosocial Variables: Perceived Self-Determination in Daily Life and Subjective Social Isolation

Two psychosocial variables were examined: perceived self-determination in daily life and subjective social isolation. Perceived self-determination in daily life was measured using a single item assessing the extent to which respondents perceived that they could freely make decisions about their lives. Respondents rated the item on a five-point scale (1–5), and the original responses were reverse-coded so that higher scores indicated greater perceived self-determination. No multidimensional self-determination scale was available in the source survey. As this measure comprised a single item, internal consistency reliability could not be assessed, and it was therefore interpreted as reflecting a narrow subjective indicator of perceived self-determination in daily life rather than as a measure of the full multidimensional self-determination construct.

Subjective social isolation was operationalized using two items assessing subjective experiences of social disconnection: feelings of loneliness and the feeling that no one knew the respondent well. Respondents rated each item on a four-point scale (1–4), and the original responses were reverse-coded so that higher scores indicated greater subjective social isolation. A composite score was calculated as the mean of the two items. The scale demonstrated good internal consistency (Cronbach’s α = 0.82). This two-item measure reflects perceived loneliness and social disconnection and should therefore be interpreted as a limited indicator of subjective social isolation rather than as a comprehensive measure of social isolation. It does not assess objective dimensions such as social network size, frequency of contact, or community participation.

#### 2.2.4. Covariates

The regression models adjusted for a set of covariates that may be associated with self-reported practical digital competence, the psychosocial variables, psychosocial well-being, or the observed associations among these variables. Demographic covariates included sex (male vs. female) and age (in years).

Socioeconomic covariates included educational attainment and household income. Educational attainment was measured on a six-point scale ranging from no formal education to graduate-level education, with higher scores indicating higher levels of education. Household income was measured as monthly household income in units of 10,000 Korean won and was log-transformed as ln(income + 1) for use in the regression models to reduce the influence of extreme values and accommodate zero household-income values.

Living arrangement was coded as living alone versus living with others. Disability-related covariates included disability type and the presence of multiple disabilities. Disability type was categorized as physical, sensory, or mental disability, with physical disability used as the reference category. The presence of multiple disabilities was coded as a binary indicator (yes/no).

Functional status and perceived health were also included as covariates in the primary adjusted specification. Functional status was assessed using instrumental activities of daily living (IADL), measured on a four-point scale (1–4), with higher scores indicating greater functional independence. Self-rated health (SRH) was measured on a five-point scale (1–5), and the original responses were reverse-coded so that higher scores indicated better perceived health. Although IADL and SRH may be associated with both self-reported practical digital competence and psychosocial well-being, their role cannot be unambiguously classified as confounding in cross-sectional data because they may also reflect consequences of disability or occupy intermediate positions within broader relationships among functional status, digital engagement, and well-being. Accordingly, the covariate set was treated as an adjustment set for estimating conditional associations rather than as a formally identified causal confounding set.

### 2.3. Statistical Analysis

Unweighted descriptive statistics were first calculated to summarize the characteristics of the analytic sample. Frequencies and percentages were reported for categorical variables, and means and standard deviations were reported for continuous variables and composite scale scores.

A regression-based cross-sectional indirect-association analysis was then conducted to examine the association between self-reported practical digital competence and psychosocial well-being and the indirect associations through perceived self-determination in daily life and subjective social isolation. The product-of-coefficients approach, commonly used in mediation analysis, was applied here to estimate cross-sectional indirect associations rather than causal mediation effects. Ordinary least squares (OLS) regression models were used for all regression equations, with heteroskedasticity-robust standard errors to account for potential heteroskedasticity in the cross-sectional survey data.

The analytical framework specified perceived self-determination in daily life and subjective social isolation as parallel psychosocial variables [29]. The two variables represent theoretically distinct dimensions of psychosocial experience, and no temporal ordering between them was imposed because the cross-sectional design could not establish such an ordering. The two specific indirect associations were estimated as the products of the regression coefficients corresponding to each psychosocial variable, and the total indirect association was calculated as the sum of the two products. The direct association between self-reported practical digital competence and psychosocial well-being was also estimated after both psychosocial variables were included in the model. The parallel specification did not assume that the two psychosocial variables were uncorrelated; both were entered simultaneously so that their associations with psychosocial well-being were estimated conditional on one another. No interaction term between the two psychosocial variables was specified because no a priori moderation hypothesis was advanced, and no serial ordering was imposed because theory and the cross-sectional design did not establish temporal precedence between them.

Four regression models were estimated. Model 1 estimated the total association between self-reported practical digital competence and psychosocial well-being, adjusting for covariates. Models 2 and 3 estimated the associations of self-reported practical digital competence with perceived self-determination in daily life and subjective social isolation, respectively. Model 4 estimated the direct association between self-reported practical digital competence and psychosocial well-being after including both psychosocial variables. All models adjusted for the covariates described in Section 2.2.4 in the primary adjusted specification. Pairwise Pearson correlations among the focal variables were examined to characterize the magnitude of their bivariate associations, and variance inflation factors in Model 4 were examined to assess potential multicollinearity. These diagnostics were not treated as formal tests of discriminant validity or common method bias.

Indirect associations were estimated using 2000 nonparametric bootstrap replications. Bias-corrected 95% bootstrap confidence intervals were constructed for the indirect association through each psychosocial variable, the total indirect association, the direct association, and the total association [29,30]. An indirect association was considered statistically significant when the corresponding confidence interval did not include zero.

The primary regression models were estimated without sampling weights. Because disproportionate allocation and post-stratification could affect regression coefficients when sampling is informative [31], we did not rely on the unweighted estimates alone. The primary adjusted indirect-association analysis was therefore re-estimated as a sensitivity analysis using the normalized final sampling weight supplied with the dataset. This survey-weight-adjusted sensitivity analysis used the same covariates and 2000 bootstrap replications as the primary analysis. Because official primary sampling unit identifiers and replicate weights were unavailable, it incorporated the final sampling weight but did not constitute fully design-based survey estimation.

To examine sensitivity to covariate specification, the primary indirect-association analysis was also re-estimated using a reduced core covariate set that excluded IADL and self-rated health while retaining demographic, socioeconomic, living-arrangement, and disability-related covariates. Additional sensitivity analyses were conducted to examine the consistency of the findings under alternative specifications of self-reported practical digital competence. These included coding ICT non-owners as 0 rather than assigning the minimum score of 1, restricting the analysis to ICT device owners only, and estimating separate models for PC competence and mobile competence among device owners. The results of these sensitivity analyses are reported in Section 3.3.

Because all focal variables were measured during the same survey interview, the ordering represented in the model was theoretically specified rather than temporally established, and reverse or reciprocal associations remain plausible. Accordingly, the findings are described in terms of associations and indirect-association patterns rather than causal pathways or mechanisms. All analyses were conducted using Stata/SE 14.2 (StataCorp LLC, College Station, TX, USA).

## 3. Results

### 3.1. Participant Characteristics

Table 1 presents the unweighted descriptive characteristics of the analytic sample. Of the 1519 adults with severe disabilities included in the analytic sample, 894 respondents were male (58.9%) and 625 were female (41.1%). The mean age was 55.1 years (SD = 18.4). The mean educational attainment score was 3.6 (SD = 1.2) on a six-point scale. The mean monthly household income before log transformation was 220.3 (SD = 349.1) in units of 10,000 Korean won. Among the respondents, 450 (29.6%) lived alone, whereas 1069 (70.4%) lived with others.

Regarding disability characteristics, physical disability was reported by 828 respondents (54.5%), sensory disability by 240 respondents (15.8%), and mental disability by 451 respondents (29.7%). Multiple disabilities were reported by 187 respondents (12.3%). The mean IADL score was 2.92 (SD = 1.10) on a four-point scale, and the mean self-rated health score was 2.58 (SD = 0.90) on a five-point scale.

Of the 1519 participants, 1109 (73.0%) reported owning the relevant ICT devices and completed the six self-reported task-ability items, whereas 410 (27.0%) were non-owners for whom these items were structurally skipped by survey design. Under the main specification, the mean self-reported practical digital competence score was 1.84 (SD = 1.02) on a four-point scale. For the psychosocial variables, the mean score for perceived self-determination in daily life was 2.96 (SD = 0.88) on a five-point scale, and the mean subjective social isolation score was 2.40 (SD = 0.64) on a four-point scale. The outcome variable, psychosocial well-being, had a mean score of 2.91 (SD = 0.76) on a five-point scale.

### 3.2. Regression-Based Indirect-Association Results

Zero-order correlations among the key study variables are presented in Table S1. Psychosocial well-being was positively correlated with both self-reported practical digital competence (*r* = 0.224, *p* < 0.001) and perceived self-determination in daily life (*r* = 0.720, *p* < 0.001), and negatively correlated with subjective social isolation (*r* = −0.432, *p* < 0.001), in the expected directions. The comparatively high correlation between perceived self-determination in daily life and psychosocial well-being indicated substantial shared variance between these two self-reported measures. Because both were measured during the same interview, common method variance cannot be ruled out as a possible contributor to this shared variance. With respect to multicollinearity, variance inflation factors in the final adjusted model were low (maximum VIF = 1.76; mean VIF = 1.33), providing no indication of problematic multicollinearity.

Table 2 presents the four adjusted regression models used in the parallel indirect-association analysis. The coefficient for the total association between self-reported practical digital competence and psychosocial well-being is denoted by c. The coefficients for the associations of self-reported practical digital competence with perceived self-determination in daily life and subjective social isolation are denoted by a_1_ and a_2_, respectively. The coefficients for the associations of these two psychosocial variables with psychosocial well-being are denoted by b_1_ and b_2_, respectively. The direct association estimated after accounting for both psychosocial variables is denoted by c′. In Model 1, self-reported practical digital competence was positively associated with psychosocial well-being after adjustment for covariates (b = 0.085, *p* < 0.001). This model explained 27.9% of the variance in psychosocial well-being.

Models 2 and 3 examined the associations between self-reported practical digital competence and the two psychosocial variables. In Model 2, self-reported practical digital competence was positively associated with perceived self-determination in daily life (*b* = 0.152, *p* < 0.001), consistent with H1. In Model 3, self-reported practical digital competence was negatively associated with subjective social isolation, but this association was not statistically significant (*b* = −0.026, *p* = 0.203, 95% CI [−0.066, 0.014]). Thus, although the direction of the coefficient was consistent with H3, the hypothesized association between self-reported practical digital competence and subjective social isolation was not supported in the adjusted model.

Model 4 estimated the direct association between self-reported practical digital competence and psychosocial well-being after including both perceived self-determination in daily life and subjective social isolation. In this model, perceived self-determination in daily life was positively associated with psychosocial well-being (*b* = 0.504, *p* < 0.001), consistent with H2, whereas subjective social isolation was negatively associated with psychosocial well-being (*b* = −0.219, *p* < 0.001), consistent with H4. After these psychosocial variables were included, the direct association between self-reported practical digital competence and psychosocial well-being was close to zero and was not statistically significant (*b* = 0.003, *p* = 0.867). The final model explained 62.6% of the variance in psychosocial well-being. The relatively high explanatory power of the final model should be interpreted in the context of the strong association and substantial shared variance between perceived self-determination in daily life and psychosocial well-being rather than as evidence, by itself, of model overfitting.

Table 3 presents the bootstrapped direct, indirect, and total associations. The specific indirect association through perceived self-determination in daily life was statistically significant (estimate = 0.076, Boot SE = 0.014, bias-corrected 95% CI [0.050, 0.106]). In contrast, the specific indirect association through subjective social isolation was not statistically significant because the confidence interval included zero (estimate = 0.006, Boot SE = 0.005, bias-corrected 95% CI [−0.003, 0.016]). The total indirect association was statistically significant (estimate = 0.082, Boot SE = 0.017, bias-corrected 95% CI [0.051, 0.115]), whereas the direct association was not statistically significant (estimate = 0.003, Boot SE = 0.017, bias-corrected 95% CI [−0.031, 0.036]). The total association between self-reported practical digital competence and psychosocial well-being was statistically significant (estimate = 0.085, Boot SE = 0.023, bias-corrected 95% CI [0.040, 0.128]).

**Figure 2:**
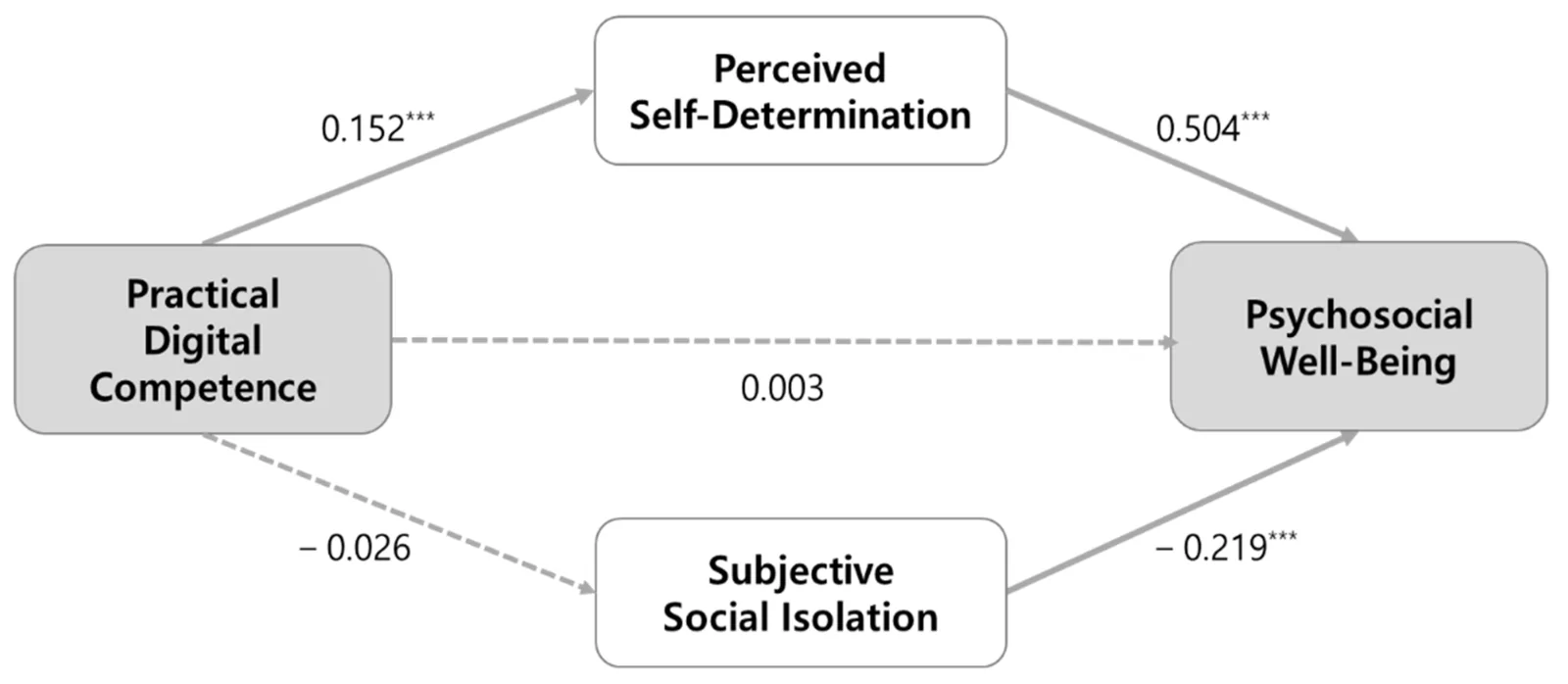
Estimated cross-sectional association model. Notes. Values represent unstandardized regression coefficients adjusted for covariates. Solid arrows indicate statistically significant adjusted regression coefficients, whereas dashed arrows indicate nonsignificant adjusted regression coefficients; the statistical significance of the corresponding product terms is reported separately in Table 3. The dashed arrow from self-reported practical digital competence to subjective social isolation reflects a nonsignificant negative association (a_2_ path), whereas the dashed arrow from self-reported practical digital competence to psychosocial well-being reflects a near-zero direct association after accounting for both psychosocial variables (c′ path); these two nonsignificant paths differ in their substantive interpretation. The ordering shown in the figure is theoretically specified and does not imply temporal or causal relationships. *** *p* < 0.001.

Taken together, the regression and bootstrap results showed a statistically significant indirect association through perceived self-determination in daily life. For subjective social isolation, self-reported practical digital competence was not significantly associated with subjective social isolation in Model 3, although subjective social isolation was negatively associated with psychosocial well-being in Model 4; the corresponding indirect association was not statistically significant in the main specification. The near-zero direct association after accounting for both psychosocial variables should not be interpreted as evidence of complete mediation or as demonstrating that perceived self-determination in daily life causally explains the association. Figure 2 summarizes the estimated cross-sectional associations and adjusted regression coefficients.

### 3.3. Sensitivity Analyses

Five sets of sensitivity analyses were conducted to examine the consistency of the main findings across alternative specifications of self-reported practical digital competence, covariate adjustment, and survey weighting (Tables S2–S6).

When ICT non-owners were coded as 0 rather than assigned the minimum score of 1 (Table S2), the indirect association through perceived self-determination in daily life remained statistically significant (estimate = 0.054, Boot SE = 0.011, BC 95% CI [0.032, 0.075]), whereas the indirect association through subjective social isolation remained nonsignificant (estimate = 0.001, Boot SE = 0.003, BC 95% CI [−0.006, 0.008]). When the analysis was restricted to ICT device owners (*N* = 1109; Table S3), the indirect association through perceived self-determination in daily life remained statistically significant (estimate = 0.081, Boot SE = 0.017, BC 95% CI [0.050, 0.116]). In this restricted sample, the indirect association through subjective social isolation was also statistically significant, although the BC confidence interval narrowly excluded zero (estimate = 0.011, Boot SE = 0.006, BC 95% CI [0.001, 0.025]).

When self-reported practical digital competence was further disaggregated into PC competence and mobile competence among device owners (Table S4), the indirect association through perceived self-determination in daily life was statistically significant for both domains (PC competence: estimate = 0.068, Boot SE = 0.016, BC 95% CI [0.037, 0.100]; mobile competence: estimate = 0.078, Boot SE = 0.017, BC 95% CI [0.047, 0.112]). The indirect association through subjective social isolation was statistically significant for PC competence (estimate = 0.015, Boot SE = 0.006, BC 95% CI [0.004, 0.030]) but not for mobile competence (estimate = 0.005, Boot SE = 0.005, BC 95% CI [−0.005, 0.017]). Across these alternative specifications of self-reported practical digital competence, the direct association remained nonsignificant, whereas the total indirect and total associations remained statistically significant (Tables S2–S4).

Two additional analyses examined sensitivity to covariate specification and survey weighting. In the reduced core model excluding IADL and self-rated health but retaining the demographic, socioeconomic, living-arrangement, and disability-related covariates (Table S5), the indirect association through perceived self-determination in daily life was statistically significant (estimate = 0.129, BC 95% CI [0.100, 0.163]). The indirect association through subjective social isolation was also statistically significant in this specification (estimate = 0.015, BC 95% CI [0.005, 0.026]). The direct association remained nonsignificant (estimate = −0.003, BC 95% CI [−0.036, 0.031]), whereas the total association remained statistically significant (estimate = 0.141, BC 95% CI [0.094, 0.186]). Both specific indirect-association estimates were larger in the reduced core model than in the primary specification that included IADL and self-rated health, although only the statistical significance of the subjective social isolation estimate changed across the two specifications.

In the survey-weight-adjusted sensitivity analysis using the normalized final sampling weight (*N* = 1519; Table S6), the indirect association through perceived self-determination in daily life remained statistically significant (estimate = 0.070, BC 95% CI [0.040, 0.100]). By contrast, the indirect association through subjective social isolation was nonsignificant (estimate = 0.004, BC 95% CI [−0.006, 0.014]). The direct association remained nonsignificant (estimate = −0.004, BC 95% CI [−0.039, 0.034]), and the total association remained statistically significant (estimate = 0.070, BC 95% CI [0.023, 0.117]).

The two parallel psychosocial variables showed different levels of consistency across the sensitivity analyses. Across all five sets of sensitivity analyses, the indirect association through perceived self-determination in daily life remained statistically significant, supporting the consistency of this indirect-association pattern across alternative specifications of self-reported practical digital competence, covariate adjustment, and survey weighting. The indirect association through subjective social isolation was not consistently observed across the main and sensitivity analyses. Specifically, it was statistically significant in the device-owner-only, PC-competence, and reduced-core specifications, but nonsignificant in the main analysis and in the non-owner-as-zero, mobile-competence, and survey-weight-adjusted specifications. Evidence for this indirect association was therefore limited and specification-sensitive and should not be regarded as conclusive. The direct association between self-reported practical digital competence and psychosocial well-being remained nonsignificant across all sensitivity analyses, whereas the total association remained statistically significant.

## 4. Discussion

### 4.1. Principal Findings

This study examined the cross-sectional associations between self-reported practical digital competence and psychosocial well-being among adults with severe disabilities in Seoul, South Korea, focusing on perceived self-determination in daily life and subjective social isolation as two theoretically distinct, parallel psychosocial variables. Perceived self-determination in daily life was assessed using a single item and is therefore interpreted throughout the Discussion as a narrow indicator of perceived autonomy or control in everyday life rather than the broader multidimensional construct of self-determination. Three principal findings emerged.

First, self-reported practical digital competence was positively associated with psychosocial well-being after covariate adjustment. The total association was modest in magnitude (b = 0.085). This association also remained statistically significant in the survey-weight-adjusted sensitivity analysis. These results suggest that perceived ability to perform everyday digital tasks may be relevant to psychosocial well-being among adults with severe disabilities, although the cross-sectional design does not establish the direction of this relationship.

Second, among the two parallel psychosocial variables, perceived self-determination in daily life showed the more consistent indirect-association pattern in analyses of self-reported practical digital competence and psychosocial well-being. Self-reported practical digital competence was positively associated with perceived self-determination in daily life, which was positively associated with psychosocial well-being. The bootstrapped indirect association through perceived self-determination in daily life was statistically significant in the main analysis (estimate = 0.076, BC 95% CI [0.050, 0.106]) and remained statistically significant across all sensitivity analyses. In absolute terms, however, the estimated indirect association was modest: a one-unit difference in self-reported practical digital competence corresponded to an indirect difference of 0.076 points in psychosocial well-being on its 1–5 scale. Its practical importance should therefore not be inferred from statistical significance alone. After accounting for both psychosocial variables, the direct association between self-reported practical digital competence and psychosocial well-being was close to zero and not statistically significant. Nevertheless, the near-zero direct association should not be interpreted as evidence of complete mediation or as demonstrating that perceived self-determination in daily life causally explains the association, because all variables were measured at the same time.

Third, self-reported practical digital competence was not significantly associated with subjective social isolation in the primary adjusted model, although subjective social isolation was negatively associated with psychosocial well-being. The corresponding indirect association through subjective social isolation was also not statistically significant in the main bootstrap analysis (estimate = 0.006, BC 95% CI [−0.003, 0.016]). Sensitivity analyses showed that this indirect association was not consistently observed across alternative coding, sample, covariate, and weighting specifications. Thus, although subjective social isolation was associated with psychosocial well-being in this sample, evidence for its indirect association in the relationship between self-reported practical digital competence and psychosocial well-being was limited, specification-sensitive, and not conclusive.

### 4.2. Interpretation and Comparison with Prior Evidence

Growing attention to the digital determinants of health has underscored that digital inequalities extend well beyond the question of internet access. Frameworks on the digital determinants of health and digital inclusion emphasize that meaningful digital inclusion requires not only access to devices and connectivity but also digital literacy and competence [1,2,3,4], together with accessible and usable service environments [32,33]. Within this broader health-equity framing, the present study operationalized self-reported practical digital competence as respondents’ perceived ability to perform specified tasks on personal computers and mobile devices, capturing a task-specific dimension of perceived digital ability rather than access or frequency of use alone. At the same time, this measure did not assess performance under standardized conditions, health-specific digital tasks, assistive technology use, or the accessibility of digital environments. The findings therefore concern a limited self-reported dimension of practical digital competence rather than comprehensive digital capability.

The positive but modest cross-sectional association between self-reported practical digital competence and psychosocial well-being, observed after adjustment for the specified covariate set, is broadly consistent with an expanding body of evidence linking digital engagement to well-being among adults with disabilities [9,15,18]. For adults with severe disabilities, who may face compounded barriers to in-person service access, mobility, and social participation, higher self-reported practical digital competence may be associated with greater opportunities or confidence to navigate health services, access information, and manage everyday tasks, all of which may be relevant to well-being [5,6,7,8,9]. This finding is consistent with health equity perspectives that locate digital competence within the broader set of conditions shaping individuals’ opportunities for autonomous participation in social and institutional life [1,2,3,4]. However, the modest magnitude of the observed association should not be interpreted as indicating a large independent contribution of self-reported practical digital competence to psychosocial well-being, and the cross-sectional analysis does not demonstrate that improving digital competence would improve well-being. Reverse or reciprocal associations remain plausible: individuals with higher psychosocial well-being may have greater opportunities, motivation, or confidence to engage with digital technologies and may consequently report higher competence.

The finding that perceived self-determination in daily life showed a more consistent indirect-association pattern in analyses of self-reported practical digital competence and psychosocial well-being is consistent with a substantial literature on the relationship between self-determination and quality of life among people with disabilities [19,20,21,22,23]. Research in this tradition has linked individuals’ sense of control, choice, and self-direction in daily life to well-being outcomes. One possible interpretation is that perceived ability to perform concrete digital tasks may be closely aligned with perceived autonomy and control over everyday activities, particularly for adults with severe disabilities whose independent participation may be constrained by functional limitations, reliance on assistance, and structural barriers. Within the specified cross-sectional model, higher self-reported practical digital competence co-occurred with higher perceived self-determination in daily life, and perceived self-determination in daily life was positively associated with psychosocial well-being. The consistently positive indirect-association pattern is therefore compatible with perspectives emphasizing autonomy and self-directed participation, but it does not demonstrate that digital competence increases perceived self-determination in daily life or that perceived self-determination subsequently increases psychosocial well-being.

Several interpretive cautions are warranted, however. The comparatively high correlation between perceived self-determination in daily life and psychosocial well-being (*r* = 0.720) also suggests substantial shared variance and possible conceptual overlap between the two self-reported constructs. Because both were measured during the same interview, common method variance may have contributed to the strength of the observed association. Accordingly, the strength of the association between perceived self-determination in daily life and psychosocial well-being, the corresponding indirect-association estimate, and the explanatory power of the final model may partly reflect shared method variance or conceptual overlap and should therefore be interpreted cautiously. Additionally, given the cross-sectional design, the observed indirect association does not establish the temporal ordering or directionality of these relationships [34].

In contrast to the perceived self-determination pattern, the indirect association through subjective social isolation was not statistically significant in the main analysis and was not consistently observed across alternative specifications. In the primary adjusted model, self-reported practical digital competence was not significantly associated with subjective social isolation (a_2_ = −0.026, *p* = 0.203), even though subjective social isolation was significantly associated with psychosocial well-being (b_2_ = −0.219, *p* < 0.001). The nonsignificant a_2_ path was therefore central to the absence of a statistically significant indirect association in the primary analysis. This pattern is broadly consistent with the mixed evidence on the relationship between digital technology use and social isolation in the existing literature [24,25,35]. While some studies have associated digital communication and online engagement with reduced loneliness and greater social connectedness [35], an evidence gap map focused on older adults found that such benefits were often limited, inconsistent, or context-dependent [25]. Because that synthesis did not specifically examine adults with severe disabilities, its relevance to the present population should be regarded as suggestive rather than directly generalizable. For adults with severe disabilities, subjective social disconnection may also be shaped by structural and relational factors, including the physical and social environment, the availability of support networks, and disability-related barriers to community participation, that are not readily addressed by digital competence alone [24,25,26]. Thus, subjective social disconnection may be relevant to well-being without necessarily varying consistently with self-reported practical digital competence. Moreover, the two-item measure may not capture other social processes through which digital competence could relate to social experience, including network structure, contact frequency, or community participation.

The sensitivity analyses further underscored the different levels of consistency of the two indirect-association patterns. The perceived-self-determination pattern remained statistically significant across alternative coding, sample, covariate, and weighting specifications, whereas the subjective-social-isolation pattern was more specification-sensitive. In particular, excluding IADL and self-rated health increased both specific indirect-association estimates and changed the statistical significance of the subjective-social-isolation estimate. Because IADL and self-rated health could represent confounding characteristics, consequences of disability, or intermediate features within broader cross-sectional relationships, neither covariate specification can be regarded as uniquely definitive. Although the subjective-social-isolation indirect association also reached statistical significance in the device-owner-restricted sample and for PC competence, these results should be interpreted descriptively rather than as evidence of a robust or established social-isolation pattern. The device-owner estimate was close to the significance boundary and was obtained from a smaller sample, while the PC and mobile analyses used the same device-owner sample and scales with similarly high internal consistency but somewhat different task content. Because no formal test of the difference between the PC- and mobile-specific coefficients was conducted, the observed differences may reflect sample composition, coding or model specification, or domain-specific task content rather than established heterogeneity.

Taken together, the contrasting patterns are compatible with the possibility that perceived ability to perform concrete digital tasks is more closely aligned with perceived autonomy or control in everyday activities than with subjective social disconnection, which may depend more strongly on relational, structural, and environmental conditions [24,25,26]. However, the greater consistency of the perceived-self-determination indirect association does not establish that perceived self-determination in daily life is a more proximal, dominant, or exclusive causal mechanism. Such an interpretation remains tentative given the cross-sectional design, the single-item measurement of perceived self-determination in daily life, and the concerns regarding shared method variance and conceptual overlap described above.

### 4.3. Implications for Practice and Policy

The present findings suggest that digital inclusion efforts targeting adults with severe disabilities may benefit from combining device access and internet connectivity with support for the practical skills needed to use digital technologies in everyday contexts. In particular, such efforts could support practical digital skills such as connecting and using external devices, changing device settings, transferring files, and creating documents. Health and welfare service navigation could be incorporated as an applied context for such training, although health-specific digital competence was not directly measured in the present study.

The more consistent indirect-association pattern involving perceived self-determination in daily life may also inform potential directions for the design and evaluation of digital competence programs. Rather than focusing exclusively on generic technical instruction, programs could instead combine technical skill development with opportunities to apply digital tools to personally meaningful goals and everyday choices. Such activities might include locating health and welfare information, managing digital documents, communicating preferences, and navigating administrative processes. Program design should be responsive to users’ own priorities and should preserve choice over whether, when, and how digital tools are used. Such autonomy-supportive approaches are consistent with broader disability research and practice emphasizing the importance of individuals’ goals, choices, and agency [19,20,21,22,23]. These recommendations should be understood as potential practice and policy directions rather than evidence that such programs would increase perceived self-determination in daily life or psychosocial well-being; these outcomes require direct evaluation in longitudinal or intervention studies.

These considerations are particularly relevant as health and welfare services increasingly rely on digital platforms [11]. For adults with severe disabilities, limited self-reported practical digital competence may compound existing barriers to accessing such services. The present findings highlight the potential importance of assessing both digital access and task-specific support needs as part of health and welfare service provision, and of designing digital service interfaces that are accessible, usable, and compatible with assistive technologies [32,33]. Training and navigation support should also be adaptable to differences in motor, sensory, cognitive, and communication needs rather than relying on a uniform delivery model. Where assistance from family members, guardians, or other support persons is needed, support should facilitate communication and task completion without displacing the preferences and agency of the person with a disability. Human-assisted digital navigation support, in which trained personnel help users access and use digital services, may also represent a meaningful complement to technological solutions and could be adapted to the needs of adults with severe disabilities [36]. Critically, the expansion of digital service delivery should not come at the cost of reducing accessible in-person alternatives; digital channels should be designed to expand rather than replace pathways to care and support.

The limited and specification-sensitive evidence for the indirect association involving subjective social isolation warrants caution in drawing practice or policy implications from this indirect-association pattern. The findings therefore do not support treating digital-skills training alone as an established intervention for loneliness or subjective social isolation. Practice and policy responses to loneliness and subjective social disconnection may therefore benefit from combining digital inclusion strategies with relational and community-based supports, such as peer connection programs, community participation initiatives, and disability-inclusive social environments. Digital communication may serve as one available form of connection for some individuals, but it should complement rather than substitute for accessible opportunities for in-person social participation and ongoing relational support. These directions are consistent with a broader social determinants perspective on psychosocial well-being but should not be interpreted as evidence of the effectiveness of specific interventions.

### 4.4. Strengths of This Study

This study has several strengths. First, it used a large sample of 1519 adults with severe disabilities residing in Seoul, South Korea, obtained from a publicly commissioned survey using stratified sampling by disability type, sex, and age. The sample size and survey design provide a substantive empirical basis for examining the associations of interest within this population. The consistency of the principal findings was also examined using the normalized final sampling weight supplied with the dataset. Second, self-reported practical digital competence was operationalized as self-reported ability to perform specific task-based activities on personal computers and mobile devices, rather than as device ownership, internet access, or self-reported frequency of use. This approach reflects a task-specific dimension of perceived digital ability that may be more closely aligned with daily life management and service navigation than broader or access-based proxies, while remaining distinct from objectively demonstrated performance. Third, this study examined two theoretically distinct psychosocial variables—perceived self-determination in daily life, reflecting perceived personal autonomy and control, and subjective social isolation, reflecting subjective social disconnection—within a parallel indirect-association framework and evaluated the consistency of the findings across alternative coding, sample, device-specific, covariate, and survey-weight specifications. This allowed a more systematic assessment of consistency and specification sensitivity than a single-model approach.

## 5. Limitations of This Study

Several limitations should be noted. The cross-sectional design precludes causal inference and does not allow for the establishment of temporal ordering among the study variables. The analyses should accordingly be interpreted as regression-based analyses of cross-sectional indirect associations rather than as tests of causal mediation; reverse or reciprocal associations—for example, in which higher psychosocial well-being facilitates greater engagement with digital activities or higher perceived digital competence—remain plausible [34]. Cross-sectional indirect-association estimates may also be biased when the theoretically specified ordering does not reflect the underlying temporal relationships; their magnitude should therefore not be interpreted as a causal mediation effect. The available dataset contained neither longitudinal follow-up nor a defensible exogenous instrument for self-reported practical digital competence; therefore, longitudinal panel or instrumental-variable approaches could not be implemented in the present study.

Additionally, all key study variables were based on self-report and collected during the same survey interview, introducing the possibility of common method bias. This concern is particularly relevant to perceived self-determination in daily life and psychosocial well-being, which were strongly correlated. Their association may therefore partly reflect shared method variance or conceptual overlap that cannot be fully separated from substantive association [37]. Although variance inflation factors did not indicate problematic multicollinearity, this diagnostic does not establish discriminant validity or rule out common method bias. The survey did not include a marker variable, independently assessed outcome, or performance-based comparator that would allow common method bias to be assessed more directly.

Beyond these common method concerns, the measurement of psychosocial constructs was subject to several additional limitations. Perceived self-determination in daily life was assessed with a single item reflecting perceived freedom and self-determination in daily life and should not be regarded as a comprehensive operationalization of self-determination as a multidimensional construct [19,20]; the findings pertaining to this indirect association are more precisely interpreted as reflecting perceived autonomy or control in everyday contexts. Subjective social isolation was measured with a two-item scale assessing loneliness and the perception of not being well known by others. Although the scale demonstrated good internal consistency (Cronbach’s α = 0.82), it does not encompass other dimensions of social isolation, such as objective isolation, social network characteristics, contact frequency, or community participation. The psychosocial well-being measure also captured positive subjective evaluations of daily life and should not be interpreted as a comprehensive measure of mental health or psychological functioning.

The self-reported practical digital competence measure, while task-based, was limited to six specific activities on personal computers and mobile devices and did not directly assess health-related digital tasks, assistive technology use, or accessibility barriers. Accordingly, the findings should not be generalized to health-specific digital competence or to individuals’ ability to navigate particular digital health services. It also represented respondents’ perceived ability rather than performance observed under standardized conditions. Among ICT non-owners, the competence items were structurally skipped and the minimum value was assigned under the main analytic coding rule. Although this was not statistical imputation, the primary coding may partly combine lack of device access with limited task competence. Thus, the primary measure should be interpreted as access-contingent and not as a pure measure of underlying digital competence.

The sampling and interview procedures also constrain interpretation and generalizability. The survey population was limited to registered, community-dwelling adults with severe disabilities residing in Seoul. It therefore excluded unregistered individuals, institutional residents, and people living outside Seoul, including rural populations who may face different digital infrastructure and service-access conditions. Because unregistered individuals may differ systematically from registered individuals in characteristics relevant to digital engagement and well-being, reliance on the disability registry as the sampling frame may introduce selection bias that cannot be evaluated with the present data. In addition, disability severity is classified within disability type in the Korean registration system and is not directly comparable across disability categories. Because the source survey did not include subgroup-specific validation or measurement-equivalence assessments, the comparability of the digital-competence and psychosocial measures across disability categories could not be established. The findings should therefore be interpreted at the overall-sample level rather than as applying uniformly across disability groups. More broadly, the relationships observed here may be shaped by the Korean context, including the disability registration and support system and the rapid digitalization of public health, welfare, and administrative services, and may therefore differ in settings with other institutional and service-delivery arrangements.

Response assistance constitutes an additional source of uncertainty. A total of 567 respondents (37.3%) received partial or full assistance from a guardian or other support person during the interview. The dataset did not indicate whether the assisting person merely supported communication or independently supplied some responses. Consequently, possible differences between unassisted and assisted interviews, including potential variation across disability and communication needs, could not be fully evaluated. Because perceived self-determination in daily life and psychosocial well-being reflect subjective evaluations, assisted responses may not always have represented respondents’ perceptions independently of the assisting person’s influence. This uncertainty may have affected the corresponding psychosocial estimates and should be considered when interpreting the observed associations. This study also did not conduct formal subgroup or interaction analyses by disability type, age, education level, functional limitation, living arrangement, interview-assistance status, or ICT ownership; the average associations reported here may therefore conceal meaningful heterogeneity.

The specification of covariates presents a further limitation. IADL and self-rated health may confound the observed associations, but they may also reflect consequences of disability or occupy intermediate positions within broader relationships among functioning, digital engagement, social disconnection, and well-being. This sensitivity to covariate specification was reflected in larger estimates for both indirect associations under the reduced-core model, with the subjective social isolation estimate changing from nonsignificant to significant. Although presenting both specifications improves transparency, the cross-sectional data cannot establish either model as uniquely definitive.

Finally, the primary analyses did not apply sampling weights. Although the survey-weight-adjusted sensitivity analysis yielded a similar substantive pattern, official primary sampling unit identifiers and replicate weights were unavailable; therefore, the weighted analysis did not constitute fully design-based survey estimation.

Several directions for future research are suggested by these limitations. Repeated-measures longitudinal designs that temporally separate assessment of self-reported practical digital competence, the psychosocial variables, and psychosocial well-being could examine whether the observed associations are better represented as parallel, sequential, or reciprocal over time. Intervention studies evaluating the effects of digital competence training—particularly autonomy-supportive programs oriented toward personally relevant goals and everyday decisions—on perceived self-determination in daily life and psychosocial well-being would complement the observational evidence presented here and help address the question of intervention effectiveness that the present design cannot answer. Such studies should assess changes in digital competence, autonomy, and well-being directly rather than assuming that the cross-sectional indirect-association pattern represents an intervention mechanism.

Future studies should also consider more comprehensive measurement instruments, including validated multidimensional self-determination scales, assessments that distinguish between subjective and objective dimensions of social isolation, and performance-based or health-specific measures of digital competence. Measures should additionally address assistive technology use, accessibility and usability barriers, digital-service design, and the distinction between device access and the ability to complete specific tasks. Where feasible, measures should also be accessible and validated across relevant disability subgroups. Combining self-report measures with observed task performance, administrative indicators, or independently assessed outcomes would help reduce reliance on a single method.

Future studies should use formal interaction or multigroup analyses to examine heterogeneity by disability, demographic, digital-access, and interview-assistance characteristics, including communication support needs and urban–rural differences. Formal tests of differences between PC-based and mobile-based competence associations, a distinction examined only descriptively in the present sensitivity analyses, would also help clarify the boundary conditions of these associations. Studies using nationally representative samples and fully specified survey-design information would further strengthen the generalizability of these findings. Including populations not covered by disability registries would also help address potential selection bias associated with registry-based sampling frames.

## 6. Conclusions

This study examined the cross-sectional associations between self-reported practical digital competence and psychosocial well-being among adults with severe disabilities in Seoul, South Korea, with indirect-association patterns estimated through two theoretically distinct, parallel psychosocial variables: perceived self-determination in daily life and subjective social isolation. Self-reported practical digital competence was positively associated with psychosocial well-being after covariate adjustment, although the magnitude of the total association was modest. The indirect association through perceived self-determination in daily life was statistically significant in the main analysis and remained statistically significant across all sensitivity analyses. The direct association was near zero after accounting for both psychosocial variables, but this should not be interpreted as evidence of complete mediation; simultaneous measurement precludes temporal or causal interpretation of this pattern. In contrast, the indirect association through subjective social isolation was not statistically significant in the main analysis and was not consistently observed across alternative specifications. Evidence for this indirect association was therefore limited, specification-sensitive, and not conclusive.

These findings suggest that digital inclusion efforts for adults with severe disabilities may benefit from combining device access and internet connectivity with support for practical digital skills relevant to everyday activities. The more consistent indirect-association pattern involving perceived self-determination in daily life highlights the potential relevance of combining technical skill development with opportunities to pursue personally meaningful goals and everyday choices. It does not, however, demonstrate that digital-skills interventions would increase perceived self-determination in daily life or psychosocial well-being. The specification-sensitive indirect-association pattern involving subjective social isolation suggests that digital competence alone may be insufficient to address subjective social disconnection and that broader relational, community-based, and environmental supports may also be relevant for this population.

The findings should be interpreted in light of several limitations. Because all focal variables were measured during the same survey interview, common method variance cannot be ruled out. The perceived self-determination measure comprised a single item, subjective social isolation was assessed using two items, and self-reported practical digital competence was not based on observed task performance. For ICT non-owners, the competence items were structurally skipped, making the primary measure access-contingent rather than a pure measure of underlying digital competence. The self-reported practical digital competence measure did not encompass health-specific digital tasks, assistive technology use, or accessibility barriers. Generalizability is limited to registered, community-dwelling adults with severe disabilities in Seoul. Repeated-measures longitudinal research is needed to clarify temporal ordering and examine whether the observed associations are better represented as parallel, sequential, or reciprocal over time. Intervention studies should evaluate whether autonomy-supportive digital-skills programs affect perceived self-determination in daily life and psychosocial well-being among adults with severe disabilities. Future research should also use validated multidimensional psychosocial measures, including measures accessible and validated across relevant disability subgroups, performance-based or health-specific assessments of digital competence, and more diverse or nationally representative samples.

## Figures and Tables

**Table 1:** Unweighted descriptive characteristics of the analytic sample (*N* = 1519).

Characteristics	Categories/Units	*n* (%) or Mean ± SD
**Sociodemographic characteristics**		
Sex	Male	894 (58.9%)
	Female	625 (41.1%)
Age	Years	55.1 ± 18.4
Education	Score (1–6)	3.6 ± 1.2
Household income ^1^	Monthly (10,000 KRW)	220.3 ± 349.1
Living arrangement	Living alone	450 (29.6%)
	Living with others	1069 (70.4%)
**Disability characteristics**		
Disability type	Physical disability	828 (54.5%)
	Sensory disability	240 (15.8%)
	Mental disability	451 (29.7%)
Multiple disabilities	Yes	187 (12.3%)
	No	1332 (87.7%)
**Health and functioning**		
IADL	Score (1–4)	2.92 ± 1.10
Self-rated health (SRH)	Score (1–5)	2.58 ± 0.90
**Digital access and competence**		
ICT device ownership	Yes	1109 (73.0%)
	No	410 (27.0%)
Self-reported practical digital competence ^2^	Score (1–4)	1.84 ± 1.02
**Psychosocial variables**		
Perceived self-determination in daily life	Score (1–5)	2.96 ± 0.88
Subjective social isolation	Score (1–4)	2.40 ± 0.64
**Outcome**		
Psychosocial well-being	Score (1–5)	2.91 ± 0.76

^1^ Household income is presented as raw monthly household income in units of 10,000 Korean won before log transformation. ^2^ Self-reported practical digital competence is presented using the main specification, in which ICT non-owners were assigned the minimum score of 1. This assignment reflects an analytic coding rule for cases in which the competence items were structurally skipped, rather than statistical imputation. Notes. Descriptive statistics are unweighted and describe the analytic sample; they should not be interpreted as population-representative estimates for adults with severe disabilities in Seoul. ICT = information and communication technology; IADL = instrumental activities of daily living; KRW = Korean won; SRH = self-rated health. Perceived self-determination refers to perceived self-determination in daily life as assessed by a single item. Subjective social isolation reflects perceived loneliness and social disconnection rather than objective social network characteristics. Higher scores indicate higher educational attainment, greater functional independence, better self-rated health, greater self-reported practical digital competence, greater perceived self-determination in daily life, and higher psychosocial well-being; for subjective social isolation, higher scores indicate greater isolation.

**Table 2:** Adjusted regression models for the cross-sectional associations (*N* = 1519).

Predictors	Model 1: PWB (Total Association, c)	Model 2: Perceived Self- Determination in Daily Life (Path a_1_)	Model 3: Subjective Social Isolation (Path a_2_)	Model 4: PWB (Direct Association, c′)
**Key independent variable**				
Self-reported practical digital competence	0.085 *** (0.022)	0.152 *** (0.027)	−0.026 (0.020)	0.003 (0.017)
**Psychosocial variables**				
Perceived self-determination in daily life (b_1_)				0.504 *** (0.022)
Subjective social isolation (b_2_)				−0.219 *** (0.024)
**Controls**				
Sex (male)	−0.084 * (0.035)	−0.073 (0.042)	0.046 (0.033)	−0.037 (0.025)
Age (years)	0.002 (0.001)	0.005 ** (0.001)	−0.002 * (0.001)	−0.001 (0.001)
Education (1–6)	0.014 (0.016)	−0.010 (0.019)	−0.006 (0.015)	0.018 (0.011)
Logged household income	0.060 ** (0.018)	0.046 * (0.019)	−0.052 ** (0.016)	0.026 * (0.012)
Living alone	−0.103 * (0.040)	0.011 (0.048)	0.233 *** (0.037)	−0.057 * (0.027)
Sensory disability	0.059 (0.047)	0.053 (0.057)	0.027 (0.046)	0.037 (0.031)
Mental disability	−0.059 (0.045)	−0.047 (0.055)	0.054 (0.043)	−0.024 (0.033)
Multiple disabilities	0.038 (0.055)	0.020 (0.066)	−0.035 (0.048)	0.020 (0.039)
IADL (1–4)	0.050 ** (0.018)	0.117 *** (0.021)	−0.043 ** (0.016)	−0.019 (0.013)
Self-rated health	0.392 *** (0.022)	0.319 *** (0.027)	−0.156 *** (0.021)	0.197 *** (0.017)
Constant	1.232 *** (0.165)	1.097 *** (0.192)	3.278 *** (0.144)	1.398 *** (0.144)
*R* ^2^	0.279	0.195	0.098	0.626
Adj. *R*^2^	0.273	0.189	0.092	0.622

Notes. Values are unstandardized regression coefficients (b) with heteroskedasticity-robust standard errors in parentheses. Model 1 estimates the total association between self-reported practical digital competence and psychosocial well-being (c). Models 2 and 3 estimate the associations between self-reported practical digital competence and the two psychosocial variables (a_1_ and a_2_, respectively). Model 4 estimates the direct association between self-reported practical digital competence and psychosocial well-being after including perceived self-determination in daily life and subjective social isolation (c′), as well as the associations of these two psychosocial variables with psychosocial well-being (b_1_ and b_2_, respectively). For the nonsignificant a_2_ path, b = −0.026, *p* = 0.203, 95% CI [−0.066, 0.014]. All covariates were entered simultaneously in each model. PWB = psychosocial well-being; IADL = instrumental activities of daily living. Logged household income was calculated as ln(income + 1). Reference categories are female, physical disability, no multiple disabilities, and living with others. * *p* < 0.05, ** *p* < 0.01, *** *p* < 0.001.

**Table 3:** Bootstrapped direct, indirect, and total associations between self-reported practical digital competence and psychosocial well-being.

Association	Estimate	Boot SE	Bias-Corrected 95% CI
Specific indirect association through perceived self-determination in daily life (a_1_b_1_)	0.076	0.014	[0.050, 0.106]
Specific indirect association through subjective social isolation (a_2_b_2_)	0.006	0.005	[−0.003, 0.016]
Total indirect association	0.082	0.017	[0.051, 0.115]
Direct association	0.003	0.017	[−0.031, 0.036]
Total association	0.085	0.023	[0.040, 0.128]

Notes. Estimates are associations based on 2000 nonparametric bootstrap replications. The specific indirect associations were calculated as a_1_b_1_ and a_2_b_2_, and the total indirect association was calculated as their sum. Because all focal variables were measured during the same survey interview, these estimates represent regression-based cross-sectional associations and should not be interpreted as causal mediation effects. Confidence intervals are bias-corrected (BC) 95% bootstrap confidence intervals. Statistical significance was evaluated based on whether the BC 95% confidence interval excluded zero. The specific indirect associations correspond to perceived self-determination in daily life and subjective social isolation, respectively. All models adjusted for sex, age, education, logged household income, living arrangement, disability type, multiple disabilities, IADL, and self-rated health. Logged household income was calculated as ln(income + 1). Boot SE = bootstrap standard error; BC = bias-corrected; CI = confidence interval; IADL = instrumental activities of daily living.

## Data Availability

Restrictions apply to the availability of these data. Data were obtained from the Seoul Welfare Foundation and are available from the corresponding author upon reasonable request and with permission of the Seoul Welfare Foundation, due to privacy and institutional restrictions.

## Supplementary Materials

The following supporting information can be downloaded at: https://www.mdpi.com/article/10.3390/healthcare14182897/s1, Table S1: Correlations among key study variables; Table S2: Sensitivity analysis of bootstrapped direct, indirect, and total associations with ICT non-owners coded as 0 (*N* = 1519); Table S3: Sensitivity analysis of bootstrapped direct, indirect, and total associations among ICT device owners only (*N* = 1109); Table S4: Sensitivity analysis of bootstrapped direct, indirect, and total associations by PC and mobile competence (*N* = 1109); Table S5: Sensitivity analysis of bootstrapped direct, indirect, and total associations in the reduced core covariate model (*N* = 1519); Table S6: Sensitivity analysis of bootstrapped direct, indirect, and total associations using the normalized final sampling weight (*N* = 1519).

## Abbreviations

The following abbreviations are used in this manuscript:

IADL	Instrumental Activities of Daily Living
ICT	Information and Communication Technology
PC	Personal Computer
SRH	Self-Rated Health
TAPI	Tablet-Assisted Personal Interviewing

## References

[1] Sieck C.J., Sheon A., Ancker J.S., Castek J., Callahan B., Siefer A. (2021). Digital inclusion as a social determinant of health. npj Digit. Med..

[2] Chidambaram S., Jain B., Jain U., Mwavu R., Baru R., Thomas B., Greaves F., Jayakumar S., Jain P., Rojo M. (2024). An introduction to digital determinants of health. PLoS Digit. Health.

[3] Richardson S., Lawrence K., Schoenthaler A.M., Mann D. (2022). A framework for digital health equity. npj Digit. Med..

[4] Arias López M.d.P., Ong B.A., Borrat Frigola X., Fernández A.L., Hicklent R.S., Obeles A.J.T., Rocimo A.M., Celi L.A. (2023). Digital literacy as a new determinant of health: A scoping review. PLoS Digit. Health.

[5] World Health Organization (2022). Global Report on Health Equity for Persons with Disabilities.

[6] Gréaux M., Moro M.F., Kamenov K., Russell A.M., Barrett D., Cieza A. (2023). Health equity for persons with disabilities: A global scoping review on barriers and interventions in healthcare services. Int. J. Equity Health.

[7] Hwang B., Shin H.-I., Chun S.-M., Park J.H. (2011). Unmet healthcare needs in people with disabilities: Comparison with the general population in Korea. Ann. Rehabil. Med..

[8] Lee S., Park H.N., Nam H.J., Kim B., Yoon J.Y. (2024). A comparison of factors associated with unmet healthcare needs in people with disabilities before and after COVID-19: A nationally representative population-based study. BMC Health Serv. Res..

[9] Manzoor M., Vimarlund V. (2018). Digital technologies for social inclusion of individuals with disabilities. Health Technol..

[10] Schalock R.L., Verdugo M.A. (2002). Handbook on Quality of Life for Human Service Practitioners.

[11] Sung W.J., Lee J. (2024). A longitudinal study on the diffusion and the divide in the use of e-government services among vulnerable citizens in Korea. Gov. Inf. Q..

[12] Hargittai E. (2002). Second-level digital divide: Differences in people’s online skills. First Monday.

[13] van Deursen A.J.A.M., Helsper E.J., Robinson L., Cotten S.R., Schulz J. (2015). The third-level digital divide: Who benefits most from being online?. Communication and Information Technologies Annual.

[14] Scheerder A., van Deursen A., van Dijk J. (2017). Determinants of Internet skills, uses and outcomes: A systematic review of the second- and third-level digital divide. Telemat. Inform..

[15] Cho M., Kim K.M. (2022). Effect of digital divide on people with disabilities during the COVID-19 pandemic. Disabil. Health J..

[16] van Deursen A.J.A.M., van Dijk J.A.G.M. (2010). Measuring Internet skills. Int. J. Hum.-Comput. Interact..

[17] van Deursen A.J.A.M., van Dijk J.A.G.M. (2011). Internet skills performance tests: Are people ready for eHealth?. J. Med. Internet Res..

[18] Duplaga M., Szulc K. (2019). The association of Internet use with wellbeing, mental health and health behaviours of persons with disabilities. Int. J. Environ. Res. Public Health.

[19] Wehmeyer M.L. (2020). The importance of self-determination to the quality of life of people with intellectual disability: A perspective. Int. J. Environ. Res. Public Health.

[20] Wehmeyer M.L., Schalock R.L. (2001). Self-determination and quality of life: Implications for special education services and supports. Focus Except. Child..

[21] Wehmeyer M.L., Schwartz M. (1998). The relationship between self-determination and quality of life for adults with mental retardation. Educ. Train. Ment. Retard. Dev. Disabil..

[22] Nota L., Ferrari L., Soresi S., Wehmeyer M.L. (2007). Self-determination, social abilities and the quality of life of people with intellectual disability. J. Intellect. Disabil. Res..

[23] Cegarra B., Cattaneo G., Ribes A., Solana-Sánchez J., Saurí J. (2023). Independent living, emotional well-being, and quality of life in people with disabilities: The mediator role of self-determination and satisfaction with participation. Front. Psychol..

[24] Gómez-Zúñiga B., Pousada M., Armayones M. (2023). Loneliness and disability: A systematic review of loneliness conceptualization and intervention strategies. Front. Psychol..

[25] Welch V., Ghogomu E.T., Barbeau V.I., Dowling S., Doyle R., Beveridge E., Boulton E., Desai P., Huang J., Elmestekawy N. (2023). Digital interventions to reduce social isolation and loneliness in older adults: An evidence and gap map. Campbell Syst. Rev..

[26] Holt-Lunstad J., Smith T.B., Baker M., Harris T., Stephenson D. (2015). Loneliness and social isolation as risk factors for mortality: A meta-analytic review. Perspect. Psychol. Sci..

[27] Seoul Welfare Foundation (2023). 2023 Survey on the Actual Conditions of Independent Living of People with Disabilities in Seoul.

[28] Ministry of Government Legislation Enforcement Rule of the Bioethics and Safety Act of the Republic of Korea.

[29] Preacher K.J., Hayes A.F. (2008). Asymptotic and resampling strategies for assessing and comparing indirect effects in multiple mediator models. Behav. Res. Methods.

[30] MacKinnon D.P., Lockwood C.M., Williams J. (2004). Confidence limits for the indirect effect: Distribution of the product and resampling methods. Multivar. Behav. Res..

[31] Solon G., Haider S.J., Wooldridge J.M. (2015). What are we weighting for?. J. Hum. Resour..

[32] Henni S.H., Maurud S., Fuglerud K.S., Moen A. (2022). The experiences, needs and barriers of people with impairments related to usability and accessibility of digital health solutions, levels of involvement in the design process and strategies for participatory and universal design: A scoping review. BMC Public Health.

[33] World Wide Web Consortium (2024). Web Content Accessibility Guidelines (WCAG) 2.2.

[34] Maxwell S.E., Cole D.A. (2007). Bias in cross-sectional analyses of longitudinal mediation. Psychol. Methods.

[35] Czaja S.J., Boot W.R., Charness N., Rogers W.A., Sharit J. (2018). Improving social support for older adults through technology: Findings from the PRISM randomized controlled trial. Gerontologist.

[36] Rodriguez J.A., Charles J.-P., Bates D.W., Lyles C., Southworth B., Samal L. (2023). Digital healthcare equity in primary care: Implementing an integrated digital health navigator. J. Am. Med. Inform. Assoc..

[37] Podsakoff P.M., MacKenzie S.B., Lee J.-Y., Podsakoff N.P. (2003). Common method biases in behavioral research: A critical review of the literature and recommended remedies. J. Appl. Psychol..

