# Supplementary material for: Practical Digital Competence and Psychosocial Well-Being Among Adults with Severe Disabilities: Cross-Sectional Indirect Associations Through Perceived Self-Determination in Daily Life
Source: Healthcare (Basel). 2026 Sep 8;14(18):2897. doi: 10.3390/healthcare14182897 (PMC13606783; doi:10.3390/healthcare14182897)
Supplement: Supplementary file 1 [file healthcare-14-02897-s001.zip › healthcare-4446787-supplementary.pdf]

Supplementary Materials

Table S1. Correlations among key study variables

Variables	1	2	3	4
1. Psychosocial well-being	–			
2. Self-reported practical digital competence	0.224***	–		
3. Perceived self-determination in daily life	0.720***	0.250***	–	
4. Subjective social isolation	-0.432***	-0.098***	-0.314***	–

Notes. Values are Pearson correlation coefficients. Only lower-triangular correlations are shown; diagonal entries are indicated by dashes. Correlations were estimated among respondents included in the analytic sample ($N = 1519$).

* $p < 0.05$, ** $p < 0.01$, *** $p < 0.001$.

Table S2. Sensitivity analysis of bootstrapped direct, indirect, and total associations with ICT non-owners coded as 0 (N = 1519)

Association	Estimate	Boot SE	Bias-corrected 95% CI
Specific indirect association through perceived self-determination in daily life	0.054	0.011	[0.032, 0.075]
Specific indirect association through subjective social isolation	0.001	0.003	[-0.006, 0.008]
Total indirect association	0.055	0.012	[0.031, 0.079]
Direct association	0.008	0.013	[-0.015, 0.034]
Total association	0.063	0.016	[0.032, 0.095]

Notes. Estimates are unstandardized associations based on 2000 nonparametric bootstrap replications. Self-reported practical digital competence was specified using the alternative coding scheme in which ICT non-owners were assigned a value of 0. For these respondents, the competence items were structurally skipped; assigning a value of 0 was an analytic coding rule rather than statistical imputation. Confidence intervals are bias-corrected (BC) 95% bootstrap confidence intervals. Statistical significance was evaluated based on whether the BC 95% confidence interval excluded zero. The specific indirect associations correspond to perceived self-determination in daily life and subjective social isolation, respectively. Because all focal variables were measured during the same survey interview, the estimates represent regression-based cross-sectional associations and should not be interpreted as causal mediation effects. All models adjusted for sex, age, education, logged household income, living arrangement, disability type, multiple disabilities, IADL, and self-rated health. Logged household income was calculated as ln(income + 1). Boot SE = bootstrap standard error; BC = bias-corrected; CI = confidence interval; IADL = instrumental activities of daily living.

Table S3. Sensitivity analysis of bootstrapped direct, indirect, and total associations among ICT device owners only (*N* = 1109)

Association	Estimate	Boot SE	Bias-corrected 95% CI
Specific indirect association through perceived self-determination in daily life	0.081	0.017	[0.050, 0.116]
Specific indirect association through subjective social isolation	0.011	0.006	[0.0009, 0.0247]
Total indirect association	0.093	0.019	[0.057, 0.134]
Direct association	-0.006	0.021	[-0.045, 0.036]
Total association	0.087	0.027	[0.036, 0.140]

Notes. Estimates are unstandardized associations based on 2000 nonparametric bootstrap replications. The analysis was restricted to respondents who reported owning ICT devices and completed all six self-reported practical digital competence items (*N* = 1109). Confidence intervals are bias-corrected (BC) 95% bootstrap confidence intervals. Statistical significance was evaluated based on whether the BC 95% confidence interval excluded zero. The specific indirect associations correspond to perceived self-determination in daily life and subjective social isolation, respectively. Because all focal variables were measured during the same survey interview, the estimates represent regression-based cross-sectional associations and should not be interpreted as causal mediation effects. All models adjusted for sex, age, education, logged household income, living arrangement, disability type, multiple disabilities, IADL, and self-rated health. Logged household income was calculated as ln(income + 1). The confidence interval for the indirect association through subjective social isolation is reported to four decimal places to indicate that the interval narrowly excluded zero. Boot SE = bootstrap standard error; BC = bias-corrected; CI = confidence interval; IADL = instrumental activities of daily living.

Table S4. Sensitivity analysis of bootstrapped direct, indirect, and total associations by PC and mobile competence (N = 1109)

Digital competence domain	Association	Estimate	Boot SE	Bias-corrected 95% CI
**PC competence**	Specific indirect association through perceived self-determination in daily life	0.068	0.016	[0.037, 0.100]
	Specific indirect association through subjective social isolation	0.015	0.006	[0.004, 0.030]
	Total indirect association	0.083	0.018	[0.047, 0.120]
	Direct association	0.009	0.019	[-0.029, 0.048]
	Total association	0.092	0.025	[0.045, 0.140]
**Mobile competence**	Specific indirect association through perceived self-determination in daily life	0.078	0.017	[0.047, 0.112]
	Specific indirect association through subjective social isolation	0.005	0.005	[-0.005, 0.017]
	Total indirect association	0.083	0.019	[0.048, 0.123]
	Direct association	-0.019	0.019	[-0.056, 0.019]
	Total association	0.064	0.026	[0.013, 0.115]

Notes. Estimates are unstandardized associations based on 2000 nonparametric bootstrap replications. PC competence and mobile competence were examined in separate cross-sectional indirect-association models among ICT device owners (N = 1109). Confidence intervals are bias-corrected (BC) 95% bootstrap confidence intervals. Statistical significance was evaluated based on whether the BC 95% confidence interval excluded zero. The specific indirect associations correspond to perceived self-determination in daily life and subjective social isolation, respectively. Because all focal variables were measured during the same survey interview, the estimates represent regression-based cross-sectional associations and should not be interpreted as causal mediation effects. All models adjusted for sex, age, education, logged household income, living arrangement, disability type, multiple disabilities, IADL, and self-rated health. Logged household income was calculated as ln(income + 1). Boot SE = bootstrap standard error; BC = bias-corrected; CI = confidence interval; IADL = instrumental activities of daily living; PC = personal computer.

Table S5. Sensitivity analysis of bootstrapped direct, indirect, and total associations in the reduced core covariate model (*N* = 1519)

Association	Estimate	Boot SE	Bias-corrected 95% CI
Specific indirect association through perceived self-determination in daily life	0.129	0.016	[0.100, 0.163]
Specific indirect association through subjective social isolation	0.015	0.005	[0.005, 0.026]
Total indirect association	0.143	0.019	[0.110, 0.182]
Direct association	-0.003	0.017	[-0.036, 0.031]
Total association	0.141	0.023	[0.094, 0.186]

Notes. Estimates are unstandardized associations based on 2000 nonparametric bootstrap replications. Self-reported practical digital competence was specified using the main coding rule, in which ICT non-owners were assigned the minimum score of 1. The reduced core model adjusted for sex, age, education, logged household income, living arrangement, disability type, and multiple disabilities; IADL and self-rated health were excluded. Confidence intervals are bias-corrected (BC) 95% bootstrap confidence intervals. Statistical significance was evaluated based on whether the BC 95% confidence interval excluded zero. The specific indirect associations correspond to perceived self-determination in daily life and subjective social isolation, respectively. Because all focal variables were measured during the same survey interview, the estimates represent regression-based cross-sectional associations and should not be interpreted as causal mediation effects. Logged household income was calculated as ln(income + 1). Boot SE = bootstrap standard error; BC = bias-corrected; CI = confidence interval; ICT = information and communication technology; IADL = instrumental activities of daily living.

Table S6. Sensitivity analysis of bootstrapped direct, indirect, and total associations using the normalized final sampling weight ($N = 1519$)

Association	Estimate	Boot SE	Bias-corrected 95% CI
Specific indirect association through perceived self-determination in daily life	0.070	0.015	[0.040, 0.100]
Specific indirect association through subjective social isolation	0.004	0.005	[-0.006, 0.014]
Total indirect association	0.074	0.017	[0.041, 0.111]
Direct association	-0.004	0.019	[-0.039, 0.034]
Total association	0.070	0.023	[0.023, 0.117]

Notes. Estimates are unstandardized associations based on 2000 nonparametric bootstrap replications. Self-reported practical digital competence was specified using the main coding rule, in which ICT non-owners were assigned the minimum score of 1. The analysis applied the normalized final sampling weight supplied with the dataset. All models adjusted for sex, age, education, logged household income, living arrangement, disability type, multiple disabilities, IADL, and self-rated health. Confidence intervals are bias-corrected (BC) 95% bootstrap confidence intervals. Statistical significance was evaluated based on whether the BC 95% confidence interval excluded zero. The specific indirect associations correspond to perceived self-determination in daily life and subjective social isolation, respectively. Because official primary sampling unit identifiers and replicate weights were not available, this weighted sensitivity analysis incorporated the final sampling weight but did not constitute fully design-based survey estimation. Because all focal variables were measured during the same survey interview, the estimates represent regression-based cross-sectional associations and should not be interpreted as causal mediation effects. Logged household income was calculated as ln(income + 1). Boot SE = bootstrap standard error; BC = bias-corrected; CI = confidence interval; ICT = information and communication technology; IADL = instrumental activities of daily living.
